# Effect of Unintentional Storage and Handling Errors of Inhaled Medications: What Does This Mean for Therapeutic Equivalence Considerations?

**DOI:** 10.1089/jamp.2018.1480

**Published:** 2019-05-24

**Authors:** Markus Wolkenhauer, Kirsten Latza, Joachim Jung, Norbert Eckhard, Frank Götzmann

**Affiliations:** Boehringer Ingelheim Pharma GmbH & Co. KG, Ingelheim am Rhein, Germany.

**Keywords:** handling, inhaled therapy, packaging, unintentional misuse

## Abstract

***Background:*** Currently, the equivalence and the substitutability of two inhaled medications are mainly driven by comparability of doses, *in vitro* performance, therapeutic equivalence and sameness, and handling of the inhalers. The packaging configuration is usually not considered as a factor.

***Methods:*** Two capsule-based inhaled tiotropium-containing products that differ by their primary packaging configurations (blister versus bottle) were compared in terms of potential handling and resulting storage errors due to unintentional misuse. Use error scenarios were identified and investigated for both the blister-packaged tiotropium and the bottled tiotropium capsules. The impact of the air exposure resulting from the packaging interaction errors was evaluated *in vitro* using fine particle dose (FPD) and delivered dose.

***Results:*** Numbers of potential errors and criticality in terms of the potential effect impact on the FPD were larger for the bottled product (between 40% and 90% loss on FPD related to initial dose). The loss of FPD could significantly impact the amount of medication that can actually reach the patient's lungs.

***Conclusion:*** When considering prescribing an inhaled medication, the specifics of the packaging and the patient's abilities and situation shall be taken into account to minimize possible handling and subsequent dosing errors.

## Introduction

Inhalation as an administration route is widely used in the treatment of chronic obstructive pulmonary disease (COPD) and asthma.^(1,2)^ Successful administration of drugs through inhalation requires adequate training of patients to enhance correct inhaler use and inhalation technique.^(2,3)^

Effective and safe use of inhalation products is dependent on their correct use.^(4,5)^ This includes, beyond the correct use of an inhaler and adequate inhalation technique, the proper preparation for use and handling of the primary packaging, that is, blisters and bottles.

In recent years, usability, the definition of critical tasks and potential use errors, has gained increasing recognition. This is reflected in new guidelines and standards regarding Design Control and Verification and Human Factors Engineering.^(6–14)^

This analysis is specifically important for new inhalation products or for switching from one inhalation product to another and should cover preparation for use, that is, from first opening of the pack to inhalation.

Even after training, usability errors can still be an issue, especially if the patient uses more than one device or is switched to another inhaler, even when the inhaler is similar and has been deemed to be interchangeable.^(5,15–17)^ A fast switch with limited training may occur specifically with the entry of generic inhalation products.^(18,19)^

Beyond inhaler handling, errors regarding the interaction with primary packaging can be an additional important source of errors.

Even for drug products that are deemed therapeutically equivalent, differences in packaging configurations that have different instructions and handling may cause patient confusion or may simply be handled incorrectly if instructions are not carefully heeded. These errors may actually impact the formulation, and therefore the effective dose, received by the patient. Thus, the dose a patient receives can depend not only on proper inhalation technique but also on product packaging and patients' ability to manage it properly.

The effectiveness of the treatment can be affected by changes in the properties of the formulation triggered by deviations from handling and storage instructions. Blister packaging is used not only for safety and convenience but also it is sometimes used to protect the drug contained within it from excessive exposure to moisture. Unlike a single bottle in which all capsules or tablets are stored in bulk, blister packaging allows only one or a limited number of capsules to be packaged in a single cavity. A potential unintentional use error for blisters can occur if one or more capsules in the compartment are exposed to ambient conditions too far in advance of use.

For bottles containing bulk capsules, a handling error can occur if the lid is not completely closed. In this case, all of the capsules contained in the bottle may be unintentionally exposed to air. In general, packaging configuration is adapted to the specific formulation, taking into account risk of exposure to air or light. Failure to do so can leave the formulation susceptible to critical errors.

The optimal range for pulmonary inhalation is generally known to be 1–5 μm in terms of aerodynamic particle size. This is measured as fine particle dose (FPD), defined as the dose of drug particles with an aerodynamic diameter <5 μm. Dry powder inhalation products are known to be sensitive to humidity. This includes both multidose dry powder inhalers (mDPIs) and the capsules that are inserted into single-dose dry powder inhalers. Manufacturers take care in the development of their product, device, and packaging to minimize the risk of exposure to humidity in the air, as exposure can significantly affect the deposition behavior and delivery performance of the inhalation product by altering the FPD and, in the case of mDPIs, causing clumping that can impede proper release of the medication.

Spiriva^®^ HandiHaler^®^ and Braltus^®^ Zonda^®^ are capsule-based dry powder tiotropium-containing inhalation products for the treatment of COPD that differ in formulation, as well as packaging configuration. The capsules for Spiriva HandiHaler contain crystalline tiotropium bromide in a lactose blend and are packed in specifically designed blister strips. The tiotropium formulation for Braltus is produced using a spray drying process^(20)^ that results in an amorphous formulation of tiotropium. Braltus capsules are packed as a 30-day supply in a high-density polyethylene (HDPE) bottle.

The instructions from the patient information leaflet for Braltus^(21)^ and Spiriva^(22)^ (approved in the EU) regarding handling and storage of the capsules are shown below.

Braltus (excerpt from section “Method of Administration/Instructions for Use and Handling”):

- Remove a Braltus capsule from the bottle immediately before use and close the bottle tightly. Place one capsule in the capsule-shaped compartment in the base of the inhaler. Do not store the capsule in the Zonda inhaler.

Spiriva (excerpt from section “Instructions for Handling and Use”):

- Remove a Spiriva capsule from the blister (only immediately before use, see blister handling) and place it in the center chamber.

Spiriva (excerpt from section “Blister handling”):

(A) Separate the blister strips by tearing along the perforation.
(B) Peel back foil (only immediately before use) using the tab until one capsule is fully visible. In case a second capsule is exposed to air inadvertently this capsule has to be discarded.
(C) Remove capsule.

Both manufacturers instruct patients to take precautions to ensure that the tiotropium capsule is not exposed to air, and the Spiriva patient leaflet also instructs patients to discard a capsule if it is inadvertently exposed and not immediately used. But what happens if patients or their caregivers fail to read these instructions, forget about them, or inadvertently expose their tiotropium capsules to air for longer than the time to prepare the treatment?

Given the different packaging for these two products, there are different potential scenarios that could lead to capsule exposure. Potential unintentional use errors were simulated *in vitro* with a risk-based approach, resulting in six potential use error scenarios for the bottle and three for the blister. In this study, these different scenarios of unintentional use errors were simulated, with FPD evaluated in each case to determine the extent of potential impact on deposition behavior of the two products.

## Materials and Methods

### Scenarios

#### Bottle

The packaging configuration of the Braltus product studied consists of a HDPE bottle with a screw lid. The bottle contains a desiccant in the lid and holds 30 capsules. The in-use shelf life is labelled as 60 days.^(21)^ Opening and closing the bottle, and any unintentional misuse of this handling step, may affect all remaining capsules in the bottle.

The following scenarios were investigated, with no other changes to the bottle, lid, or desiccant.

(1) Lid closed to first resistance—Lid screwed on bottle until first resistance, but not tightened, and capsules are stored as bulk within the bottle.
(2) Lid loosely closed—Lid screwed on bottle, and capsules are stored as bulk within the bottle.
(3) Lid put on top—Lid sits on top of the bottle (without screwing), and capsules are stored as bulk within the bottle.
(4) Open storage—Capsule openly stored on flat surface outside the bottle.
(5) Pill box—Capsules stored in pill box (five compartments).
(6) Open bottle—Lid completely removed, and capsules are stored as bulk within the open bottle.

Scenario 1 simulates patients with limited ability to apply the force necessary to close the lid. In this simulation, a patient would screw on the lid until they feel a resistance, but do not tightly close it. The difference between feeling first resistance and tightly closing the lid is only a few degrees of turning. In the second and third scenarios, patients or caregivers are not careful in closing the lid. In Scenario 2, the lid is screwed only several turns on the bottle, and in Scenario 3, the lid sits only loosely on the bottle. To ensure consistent conditions in Scenario 2, the lid is closed to first resistance and then turned one revolution back.

In Scenario 4, a capsule is removed from the bottle and left in the open air. This may occur when a patient or a caregiver takes out a capsule but the capsule is not immediately used, either because of daily routine (e.g., preparing medication and then having breakfast or washing first) or because the process is spontaneously interrupted (e.g., a telephone call). Scenario 5 simulates preparing the medication beforehand by the caregiver or the patient. In this case, the capsules are removed from the bottle and placed in pill boxes with five compartments. In Scenario 6, a patient or caregiver simply forgets to close the bottle (e.g., the lid is misplaced or left off).

#### Blister

The blister packaging configuration for Spiriva HandiHaler capsules consists of six blister strips, each with one cavity that holds five capsules and is sealed with foil (packaging configuration for Europe). Thus, a monthly pack consists of 30 capsules. The labelled in-use shelf life is 30 days. The following scenarios were investigated:

(A) Open storage—Capsule left on flat surface outside the blister.
(B) Pill box—Capsules stored in pill box (five compartments).
(C) Open blister—Cover foil completely removed, exposing the remaining capsules stored inside the blister.

Scenario A for the blister packaging (capsules are left in open air) is comparable to Scenario 4 for the bottle. Scenario B (capsules stored in a five-compartment pill box) is comparable to Scenario 5 for the bottle. Scenario C is comparable to Scenario 6 for the bottle, where the blister foil is completely removed from the strip, exposing the capsules remaining in the packaging. All scenarios are applicable for separate, individually packed capsules (e.g., Spiriva US packaging configuration).

### Setup of experiments

#### Storage, timing, and sampling

The experiments were all conducted under conditions of 25°C/60% relative humidity (climate zone II). Testing and storage time were conducted according to in-use shelf life (30 or 60 days, as per label) or until the normalized FPD (NFPD) reached a stable minimum. Each scenario was tested at 0, 1, 2, 4, 8, 24, and 48 hours, then every second/third day for the duration of the experiment. Two batches were used for both Braltus and Spiriva. Five capsules were used from different bottles/blisters per batch and at different time points in each scenario. The testing parameter used was FPD using a short stack Anderson Cascade Impactor (ssACI). The NFPD is reported, normalization being relative to the FPD at *t* = 0.

#### Materials

The capsules used were from Braltus+Zonda Inhaler (Teva, Batches LC29738/LC30122 [nominal dose: 13 μg tiotropium cation]) and from Spiriva+HandiHaler (Boehringer Ingelheim, Batches 607900/608564 [nominal dose: 18 μg tiotropium cation]).

### Methods

A Flow Control Unit (Boehringer Ingelheim) that conforms to both US Pharmacopeia (USP) and European Pharmacopoeia (EP) standards was used for all of the experiments. An ssACI (consisting of a sample induction port [SIP], Preseparator, Stage 0, Stage 1, Filter) (Copley) was used to test FPD. Aerodynamic particle size distribution measurements were carried out using a next-generation impactor (Copley). Delivered-dose tubes were used according to USP/EP (Boehringer Ingelheim). In all experiments, the flow rates were set according to a pressure drop of 4 kPa, which were identical for both inhalers.

High performance liquid chromatography (HPLC) and ultra-performance liquid chromatography were conducted using the Agilent 1200 SL with Diode-array Detector, the Agilent 1200 SL Multiple Wavelength Detector, and Waters Acquity Tunable UV Detector. All storage took place in a climate storage chamber (Weiss).

#### Normalized fine particle dose

The FPD was measured using an abbreviated ssACI, which consists of a SIP, a USP high top (connection between the SIP and preseparator), a preseparator, separation stages 0 and 1 with collection plates, and a final filter. The respective inhalers were connected to the induction port of the impactor through separate adapters. The ssACI was connected to flow control equipment in accordance with EP/USP.

Before the experiments, the preseparator and collection plates were coated with a glycerol/Brij 35 mixture, and the ssACI was tested for tightness. The aerodynamic FPD was defined as the active ingredient dose with a particle size <5 μm, and it was calculated to take into account the different cutoff diameters of the separation stages due to the different flow rates and extrapolation to 5 μm. The flow rate and suction time were set to meet the requirements of a pressure drop of 4 kPa and 4 L volume of air passing the inhaler according to USP/EP standards.

The two test products have different nominal doses, as well as different storage condition scenarios and storage times (up to 30 or 60 days). Furthermore, inter- and intrabatch variabilities will lead to different FPD. Therefore, to compare the two products, the FPD results were normalized to the initial FPD (*t* = 0) in all of the experiments to ensure the same starting point. Thus all data are related to the FPD (*t* = 0) of capsules used correctly without misuse. Thus, despite differences in individual batches, intrabatch variability, and nominal doses, the change over time can be truly and easily compared. FPD results are therefore reported and discussed as NFPD.

#### Delivered dose

The delivered dose (DD) or emitted dose is the total amount of drug emitted from the inhaler device and therefore available to the inhaler user. In this experiment, DD was measured to determine if, beyond FPD, the actual amount of drug available to the patient from the inhaler was impacted by inadvertent exposure of the capsule to air. If the amount of drug changed, this might suggest some degree of degradation of the active pharmaceutical ingredient (API).

The apparatus and methods were based on current EP and USP, consisting of flow control unit and sample collection tubes. The outlet of the sample collection tube had an open-mesh screen supporting a glass-fiber filter. The flow rate and actuation time were set to meet the requirements of a pressure drop of 4 kPa and 4 L volume of air passing the inhaler according to EP/USP by adjusting the pressure drop through the inhaler. After adjusting the flow rate, 10 capsules were measured individually. To extract and determine the active ingredient content, each sample collection tube was rinsed with an appropriate volume of diluent. Each aliquot was measured using HPLC.

## Results

### Bottle

**FIG. 1.**
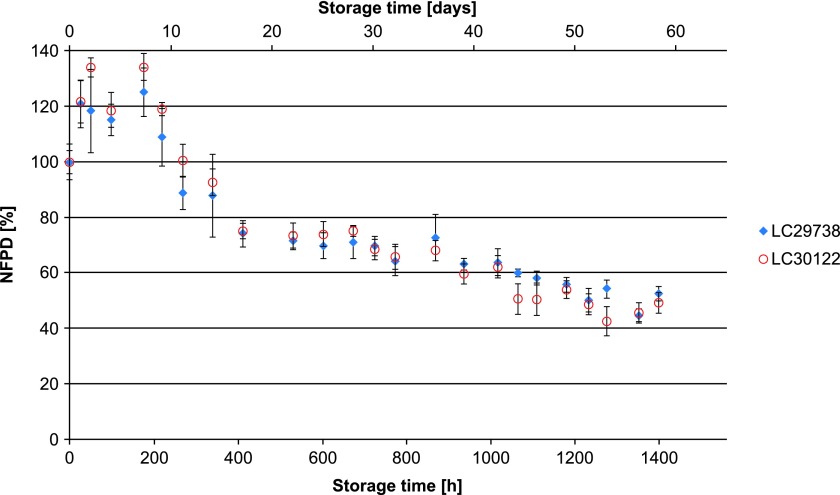
Change in fine particle dose relative to start value over storage time in Scenario 1 for the bottle configuration (lid closed to first resistance).

**FIG. 2.**
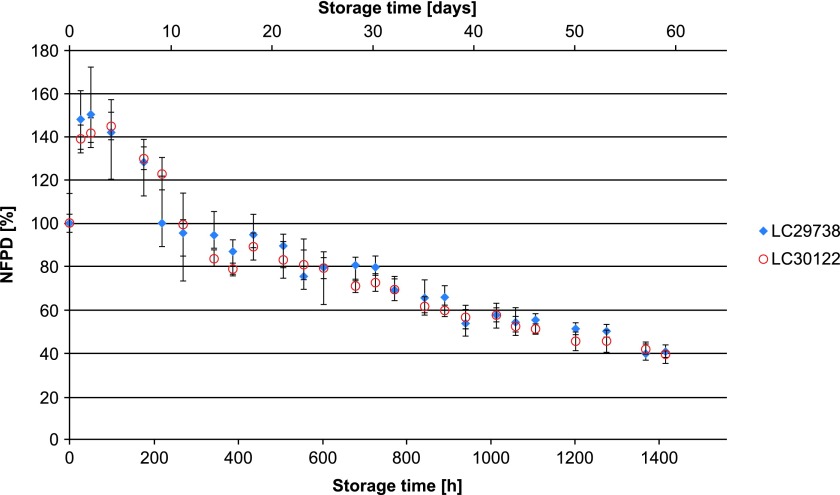
Change in fine particle dose relative to start value over storage time in Scenario 2 for the bottle configuration (lid loosely closed).

**FIG. 3.**
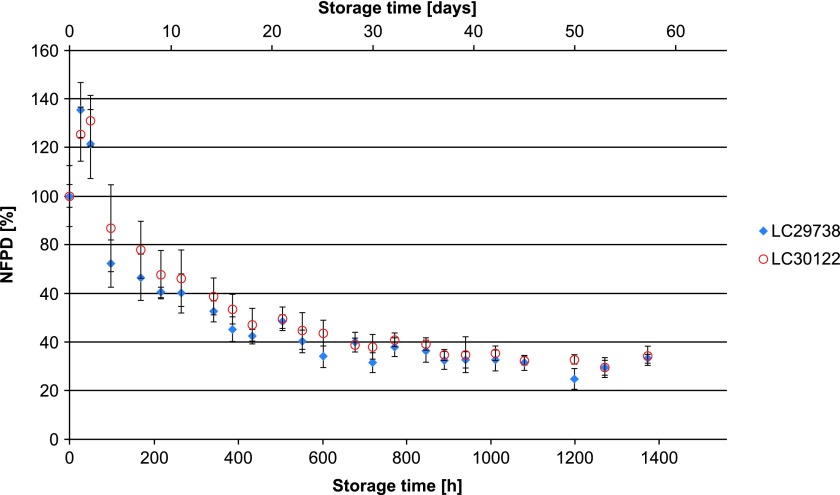
Change in fine particle dose relative to start value over storage time in Scenario 3 for the bottle configuration (lid put on top).

**FIG. 4.**
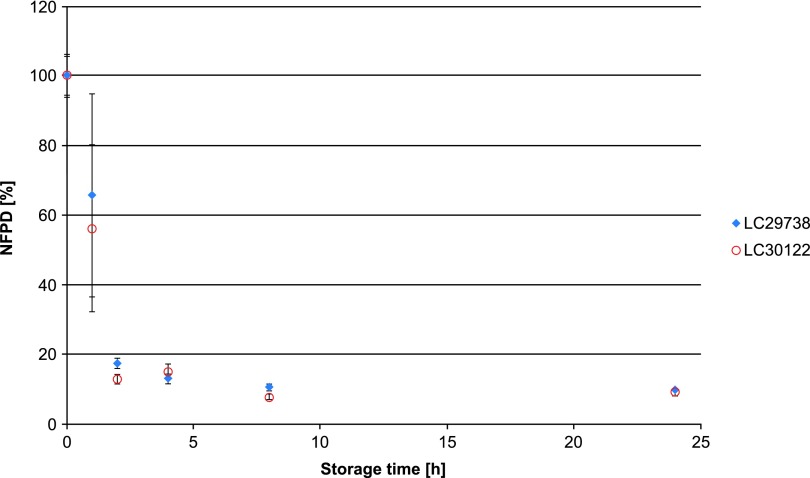
Change in fine particle dose relative to start value over storage time in Scenario 4 for the bottle configuration (capsule stored in open).

All experiments were carried out using two different batches of the drug product, which in all cases yielded similar results. In Scenario 1 (lid closed to first resistance but not tightened), the NFPD initially increased and then decreased until the end of the in-use time of 60 days to ∼50% of the initial dose (Fig. 1). Scenario 2 (lid loosely closed with screwing) showed similar behavior: the NFPD decreased to ∼40% of the initial dose (Fig. 2). For Scenario 3 (lid put on top of the bottle without screwing), the NFPD dropped below 40% of the initial dose at the end of the in-use time (Fig. 3). Scenario 4 (capsule stored in the open on a flat surface) revealed a sharp decrease in NFPD within 2 hours to a NFPD below 20% of the initial dose and after 8 hours to lower than 10% (Fig. 4).

**FIG. 5.**
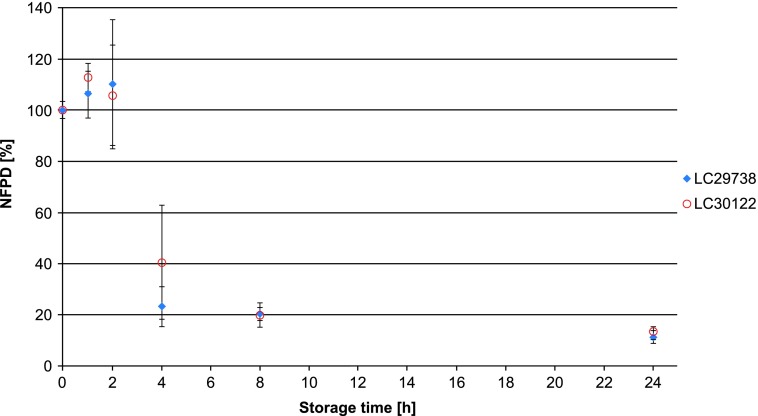
Change in fine particle dose relative to start value over storage time in Scenario 5 for the bottle configuration (capsules stored in pill box).

**FIG. 6.**
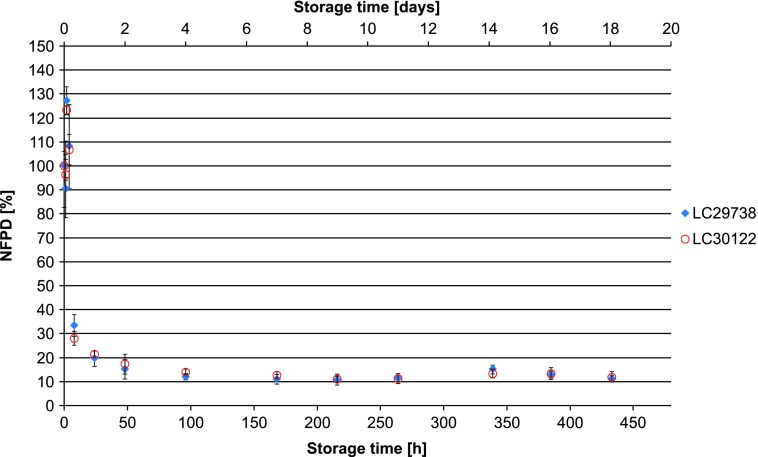
Change in fine particle dose relative to start value over storage time in Scenario 6 for the bottle configuration (bottle open).

In Scenario 5 (capsules stored in pill box), the mean NFPD remained stable for 2 hours, followed by a sharp decrease to 20% and 40% in the two batches, with high variability. After 8 hours, the NFPD was ∼20% for both batches and decreased even further after 24 hours to about 13% (Fig. 5). Scenario 6 (storage in an open bottle and lid completely removed) showed a decrease of the dose to about 20% of the initial dose within 24 hours, with a further decrease of the FPD to ∼13% after 7 days (Fig. 6). Values are listed in Table 1.

**Table 1.** Normalized Fine Particle Dose at Different Time Points in Experiments of Bottle Packaging Configuration

	*Batch*	*Time (hours)*	*Time (days)*	*NFPD (%)*
Scenario 1 (lid closed to first resistance)	LC29738	1399	58	52.5
	LC30122	—	—	49.3
Scenario 2 (lid loosely closed)	LC29738	1415	59	40.7
	LC30122	—	—	39.4
Scenario 3 (lid put on top)	LC29738	1374	57	33.2
	LC30122	—	—	34.5
Scenario 4 (open storage)	LC29738	1	—	65.7
	LC30122	—	—	56.1
	LC29738	2	—	17.2
	LC30122	—	—	12.8
	LC29738	24	1	9.8
	LC30122	—	—	9.1
Scenario 5 (pill box)	LC29738	4	—	23.2
	LC30122	—	—	40.5
	LC29738	8	—	20.3
	LC30122	—	—	19.8
	LC29738	24	1	11.2
	LC30122	—	—	13.3
Scenario 6 (open bottle)	LC29738	8	—	33.5
	LC30122	—	—	28.0
	LC29738	24	1	19.6
	LC30122	—	—	21.4
	LC29738	48	2	15.2
	LC30122	—	—	17.4
	LC29738	96	4	12.0
	LC30122	—	—	14.0
	LC29738	168	7	10.9
	LC30122	—	—	12.8
Open/close (open bottle)	LC29738	24	1	18.7
	LC30122	—	—	16.9
	LC29738	48	2	11.9
	LC30122	—	—	13.4

NFPD, normalized fine particle dose.

**FIG. 7.**
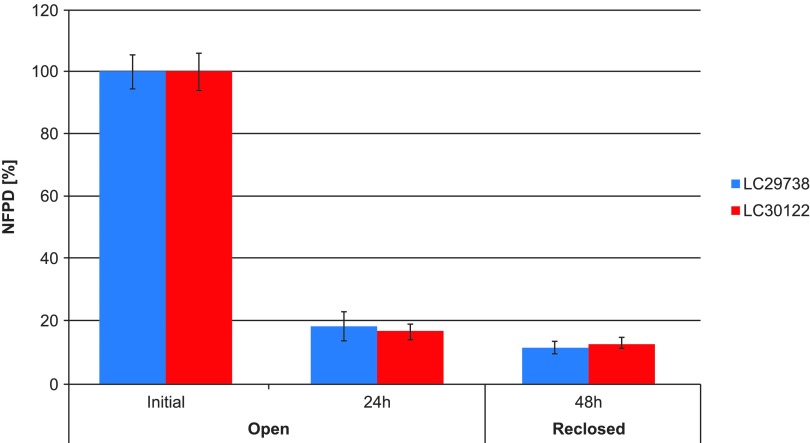
Change in fine particle dose relative to start value over storage time of open bottle: initially, after 24-hour storage in opened bottle and after 48 hours (reclosing the bottle after 24-hour open storage and further 24-hour storage in the then closed bottle).

For Scenario 6 (open bottle), reversibility of the decrease in NFPD was also tested. The bottle, after having been open for 24 hours, was closed again and analyzed after an additional 24-hour storage for DD and FPD. The NFPD remained unchanged, so the reduction in FPD observed after the first 24-hour exposure was not recovered (Fig. 7).

**FIG. 8.**
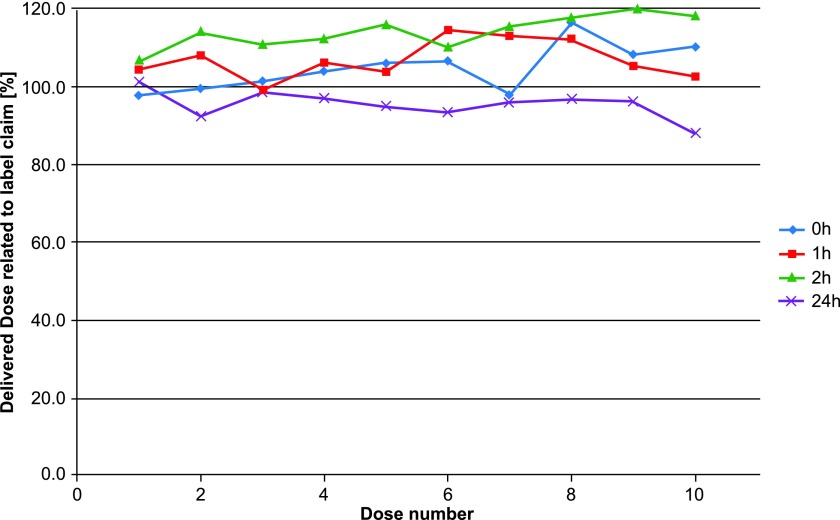
DD related to label claim (DD = 10 μg/capsule) after 0, 1, 2, and 24-hour open storage. DD, delivered dose.

**FIG. 9.**
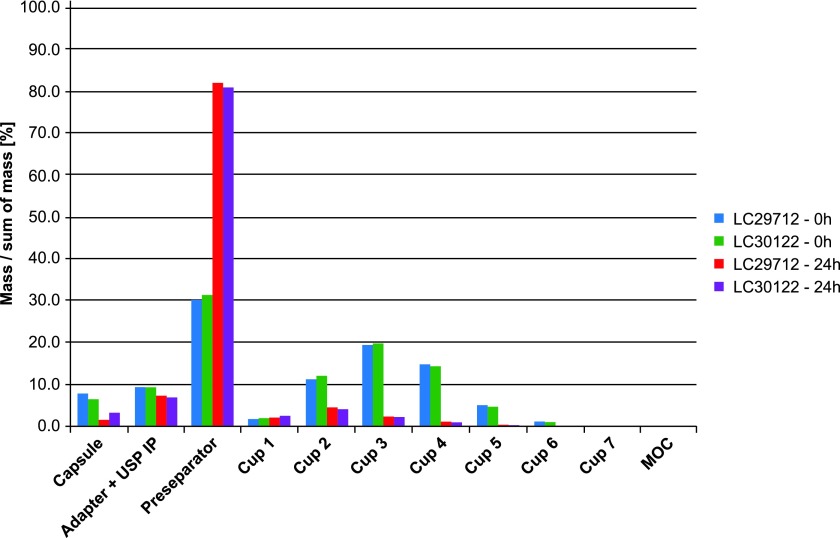
Aerodynamic particle size distribution.

The DD—the total active ingredient dose available from the inhaler—was not affected with storage of 24 hours (Fig. 8). This is an indicator that changes toward larger particle sizes caused the drop and not any degradation of the API. To confirm this, the aerodynamic particle size distribution was measured initially and after 24 hours. After 24-hour storage, the dramatic increase of particle mass present in the preseparator and the decrease in the subsequent compartments confirm this hypothesis (Fig. 9). Values are listed in Table 2.

**Table 2.** Delivered Dose Related to Label Claim (Delivered Dose = 10 μg/Capsule) at 0, 1, 2, and 24 Hours of Bottle Packaging Configuration

*Capsule No.*	*0 Hour (%)*	*1 Hour (%)*	*2 Hours (%)*	*24 Hours (%)*
1	97.9	104.4	106.6	101.3
2	99.7	108.2	114.3	92.5
3	101.4	99.3	111.1	98.7
4	104.1	106.3	112.4	97.3
5	106.2	104.0	116.1	95.0
6	106.8	114.6	110.4	93.5
7	97.9	113.3	115.6	96.1
8	116.7	112.3	118.0	97.0
9	108.3	105.4	120.1	96.5
10	110.4	102.8	118.3	88.1
Average	104.9	107.1	114.3	95.6

### Blister

**FIG. 10.**
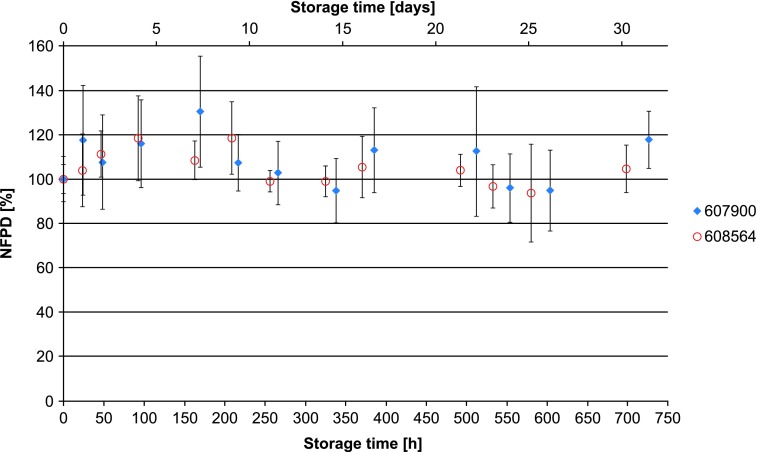
Change in fine particle dose relative to start value over storage time in Scenario A for the blister configuration (capsules stored in open).

**FIG. 11.**
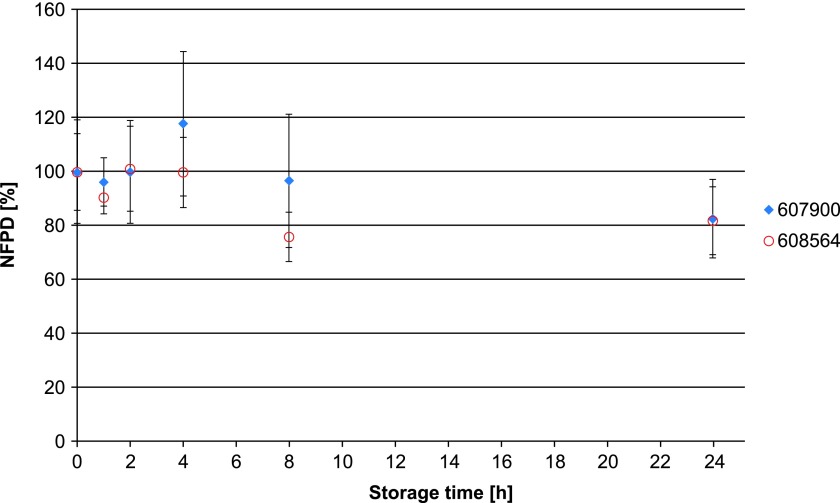
Change in fine particle dose relative to start value over storage time in Scenario B for the blister configuration (capsules stored in pill box).

**FIG. 12.**
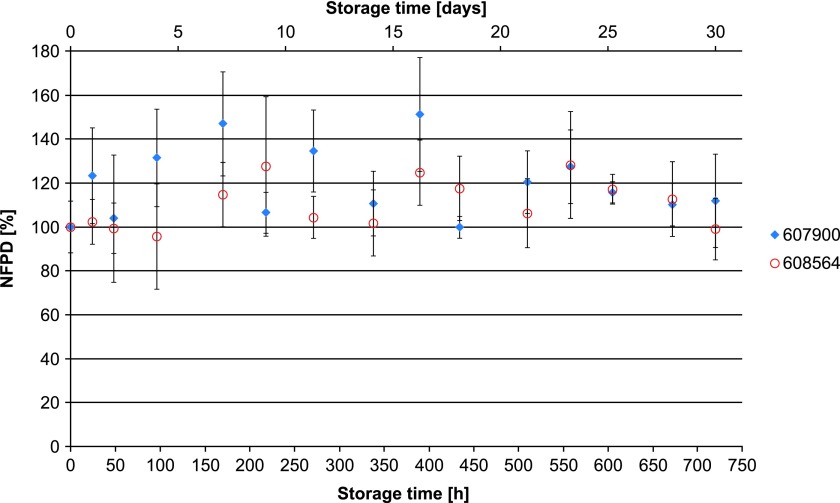
Change in fine particle dose relative to start value over storage time in Scenario C for the blister configuration (capsules stored in blister, but the foil is removed from the strip).

The NFPD for Scenario A (open storage) showed no decrease and remained largely constant in the range of 80%–120% of the initial value over the storage time of 30 days (Fig. 10). In Scenario B (pill box), the same was observed over a storage time of 24 hours, with an NFPD at 30 days of about 82% (Fig. 11). For Scenario C (open blister), the NFPD at the end of the storage time of 30 days was unchanged (∼100% of the initial dose) and showed no decrease during storage (Fig. 12). Values are listed in Table 3.

**Table 3.** Normalized Fine Particle Dose at End of Experiments of Blister Packaging Configuration

	*Batch*	*Time (hours)*	*Time (days)*	*NFPD (%)*
Scenario A (open storage)	607900	726	30	117.8
	608564	726	30	104.6
Scenario B (pill box)	607900	24	1	82.6
	608564	24	1	82.0
Scenario C (open blister)	607900	720	31	111.9
	608564	720	31	99.0

## Discussion

The dose, and therefore the effect, of an inhaled medication is dependent on correct use, which is described in the package leaflet. FPD is tested *in vitro*, and efficacy and safety are verified in clinical studies. Human factor studies address potential use errors and risk mitigation, but usually do not include the potential impact of packaging use errors on product performance. This study addresses the impact of potential use errors in the handling of primary packaging on the formulation and resulting FPD dose of two different tiotropium powder capsule formulations provided in a blister or bottle.

A decrease in FPD was observed in all unintentional use error scenarios of the bottled medication. The extent of the decrease in NFPD was dependent on the type of use error, and this decrease in NFPD performance was not reversible.

Two different types of use error scenarios were tested: closed (lid closed to first resistance, lid loosely closed, and lid put on top) and open (open bottle, pill box, and open storage). The closed use error scenarios led to a decrease in the NFPD over the 60-day in-use time of 40%–60%.

Factors that could influence this type of error are the patient's abilities and strength, as well as simply neglecting to properly close the bottle. For the capsules stored in the bottle, the open scenarios show a rapid decrease in NFPD of 10%–20% of the initial dose within 24 hours. For the scenario in which the capsule was left in the open air, a decrease in the NFPD reached between 10% and 20% in 2 hours; for the pill box scenario, the NFPD was at about 20% of the original dose after 8 hours. This type of error can be caused by daily routine, preparing medication hours before the application, or simply by leaving the bottle open.

These use errors are judged critical as, first, it may not be discovered by the patient or caregiver, and, second, they are not reversible. The unintentional use error scenarios for the blister-packed tiotropium revealed no major drop in NFPD for any of the scenarios over the 30-day in-use time for the open blister and open storage scenario and for 24 hours in the pill box scenario.

The bottled tiotropium product showed a decrease in NFPD across all of the scenarios, with even the lid tightened to first resistance showing a loss of about 50% of the original FPD over the 60-day in-use time of the capsule.

The experiment did demonstrate that the DD—the amount of active ingredient—did not change despite a significant decrease in FPD. This suggests that shifts toward larger particle sizes and not degradation of the active drug caused the decrease in FPD. Thus, even though the active drug appears to not be significantly impacted by inadvertent exposure to air over 24 hours, the size of the particles is affected for these bottle-packed capsules, and the decrease in FPD is not reversible.

So, a patient who does not close the bottle correctly or routinely uses a pill box to manage adherence to the Braltus tiotropium product risks receiving only a fraction of the initial dose throughout the entire treatment. Likewise, if a patient or caregiver leaves a full bottle open with no lid on for only 24 hours, a patient may only receive a fraction of the initial dose for the rest of the package up to 29 days of treatment.

The tiotropium formulation of the blistered product showed a higher resistance against the effects of air exposure compared with the bottle-packed product. However, unlike with the bottled product, the manufacturer still instructs the patient to discard a capsule that is inadvertently exposed and not immediately used. It is interesting to note that about 18% of FPD was lost over 24 hours when the Spiriva tiotropium capsule was stored in a pill box. There may be other factors that impact the difference, such as capsule material or the different formulations of tiotropium, but these factors were not explored in this study.

The impact of potential unintentional use errors associated with primary packaging materials for the bottle versus blister packaging demonstrates major differences that could have significant impact on the NFPD. This underscores the importance of testing inhalation maneuvers and preparation for use in human factor studies and suggests an important link between packaging-related use errors and the quality and integrity of an inhalation product.

If a patient or caregiver receives neither an indication that an error has been committed nor any information on what consequences that error may have on the therapeutic dose, they will be unaware of the possible negative impacts on treatment.

## Conclusion

This study demonstrates large differences in the effects of unintentional use errors related to different packaging configurations—bottle versus blister—for two tiotropium-containing products that have been deemed to be therapeutically equivalent in Europe. Comparing the packaging configurations in a risk-based approach, the number of possible scenarios for unintentional use errors for the bottle configuration (*N* = 6) is higher than for the blister (*N* = 3).

The NFPD in the open storage scenarios for the bottle-packed product (capsule left open, pill box, and open bottle) dropped to around 10%–20% of the initial dose within 2–24 hours, whereas the blister-packed product only demonstrated a drop of ∼80% within 24 hours in the pill box scenario.

Since it is not mentioned in the bottle product patient information leaflet, and no alteration of the medication is visible, the patient or caregiver has no indication that a use error took place or what the consequences may be and are unaware of the possible negative impacts on their treatment and health status. Conversely, in the patient information leaflet of the blister medication, for risk mitigation it is stated that in case of an erroneous exposure of a second capsule by pulling the blister foil too far, the second capsule has to be discarded; however, there is nothing similar for the bottled medication. Potential use errors that may impact the quality of the product should be clearly highlighted on the label, particularly when the use error is not obvious or may result in inadequate medication.

Currently, the judgment of therapeutic equivalence is mainly driven by comparability of doses and handling of the inhalers.^(23)^ The primary packaging system appears to be of less consideration as far as therapeutic equivalence is concerned. This may be justifiable for inhalers in which the drug is an integral part. In such cases, the inhaler only needs to be removed from the packaging once and the patient does not have to handle individual dose containers.

However, for single-dose DPIs that require loading of individual doses by taking them out of a primary packaging on a daily basis, correct handling of the primary packaging system for the individual dose containers is a critical task and an essential prerequisite for correct use. This study shows that considering only the dose and the inhaler for equivalence is not sufficient. It is essential to consider the full packaging system as well to avoid possible handling errors and medication failures.

## Notes

Reviewed by:

Myrna Dolovich
